# Endometriosis presenting as a rectal stricture in a patient with ulcerative colitis and primary sclerosing cholangitis: a case report

**DOI:** 10.3389/fgstr.2025.1584899

**Published:** 2025-05-09

**Authors:** Daniel Chorley, Ibrahim Mian, Deloshaan Subhaharan, Pradeep Kakkadasam Ramaswamy

**Affiliations:** ^1^ Department of Digestive Health, Gold Coast University Hospital, Gold Coast, QLD, Australia; ^2^ Department of Health Science and Medicine, Bond University, Gold Coast, QLD, Australia

**Keywords:** ulcerative colitis, endometriosis, stricture, primary sclerosing cholangitis, inflammatory bowel disease, colorectal cancer, cancer surveillance, colectomy

## Abstract

Patients with ulcerative colitis (UC) are at an increased risk of colorectal cancer (CRC), which is compounded in the presence of other risk factors such as primary sclerosing cholangitis (PSC) and stricture. We report a middle-aged lady who developed a rectal stricture on the background of ulcerative colitis and primary sclerosing cholangitis. Although initial endoscopic biopsies showed no dysplasia, the patient proceeded with a total colectomy due to the concern for an underlying malignancy. Histology of the rectal specimens revealed endometriosis at the site of the stricture. This case emphasises the importance of evaluating the risk factor profile for malignancy in patients with ulcerative colitis while also highlighting the need to consider alternative aetiologies for rectal strictures.

## 1 Introduction

Inflammatory bowel disease (IBD) increases the risk of developing colorectal cancer (CRC) (1). There is a two-fold risk of CRC in patients with ulcerative colitis (UC) compared with the general population and contributes to 10-15% of the annual deaths in IBD patients (1). Current guidelines recommend screening colonoscopy to be performed 8 years from symptom onset in patients with left-sided colitis or pancolitis, or at diagnosis in patients with PSC. In patients with high-risk features including previous stricture, previous dysplasia, PSC/transplant for PSC, family history of CRC in a first-degree relative < 50 years of age, annual surveillance colonoscopy is recommended (1, 2).

Colonic strictures represent abnormal narrowing within the bowel secondary to scar tissue formation from repetitive cycles of active inflammation and healing. They are not exclusive to IBD and can arise from a variety of alternative causes including endometriosis, whereby endometrial tissue is found outside the uterus (3). In this article, we present a rare case of an endometriotic rectal stricture initially concerning for malignancy in a patient with UC and PSC.

## 2 Case description

A woman in her early 40’s was seen in the Inflammatory bowel diseases clinic for routine assessment. She was diagnosed with UC pancolitis in 1999 and with large duct PSC in 2022 confirmed on magnetic resonance cholangiopancreatography. She had no known history of endometriosis and had no other past medical history. She reported occasional abdominal pain and bloating, her bowels opened daily without any diarrhoea or blood. Her menstrual cycle was of normal length with infrequent menorrhagia and minor dysmenorrhoea. She had one child conceived naturally. Her current medications included vedolizumab four weekly, 6-mercaptopurine, and mesalazine. Vedolizumab was commenced in early 2022 with dose escalation in late 2022 due to ongoing progressive disease activity and evidence of a rectal stricture on endoscopy. Prior to this she was managed with steroids and 6-mercaptopurine. She unfortunately developed steroid dependency developing complications of steroid induced osteoporosis. She had an unknown reaction to azathioprine. She denied smoking or alcohol and her family history was unremarkable for colorectal cancer. Her vital signs were normal with a benign abdominal examination.

### 2.1 Investigations

**Figure 1:**
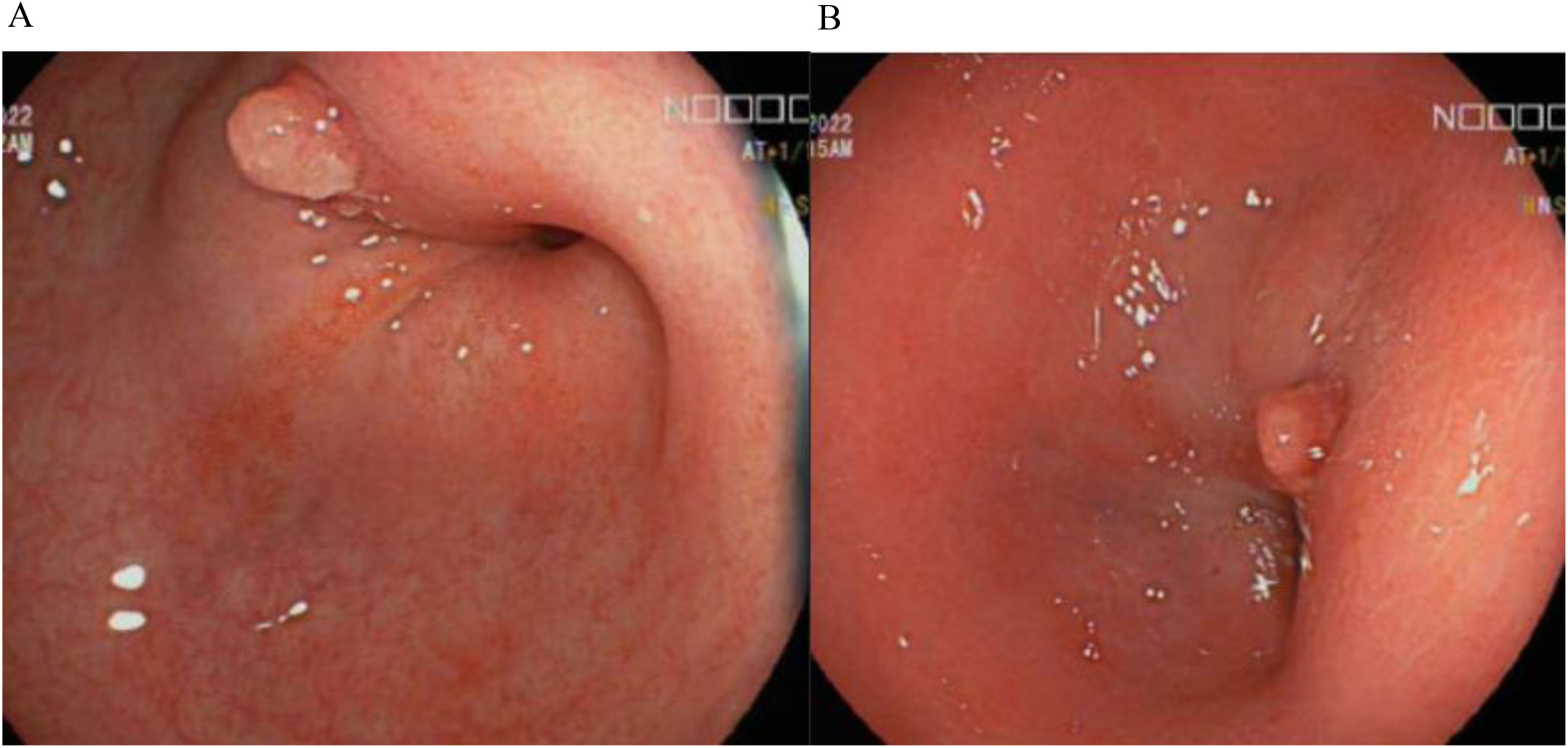
**(A, B)** Endoscopic images of the patients stricture at the recto-sigmoid junction.

Her biochemical results were unremarkable with haemoglobin 124g/L, C-reactive protein 6.9mg/L (N = <5mg/L), albumin 36g/L and faecal calprotectin 52ug/g. Colonoscopy in 2022 demonstrated a rectosigmoid stricture (**Figures 1A, B**) which was recurrently biopsied and dilated over the next 18 months, however repeated histology did not demonstrate any evidence of dysplasia. Disease activity at time of colonoscopy showed confluent inflammation in the ascending colon and caecum ulcerative colitis endoscopic index of severity (UCEIS) score of 3, patchy inflammation in the transverse colon (UCEIS 2) and patchy inflammation in the sigmoid and descending colon (UCEIS 3-4).

### 2.2 Differential diagnosis

The differential diagnosis included both malignant and benign strictures. Given her risk factor of PSC and persistence of the rectal stricture, malignancy was highly concerning even though prior superficial biopsy samples were negative. Benign stricture secondary to IBD was also considered. Other causes of benign strictures including diverticulosis, ischaemia, radiation, and endometriosis were considered less likely given no history of the same and investigations to date were unremarkable for these.

### 2.3 Therapeutic intervention

**Figure 2:**
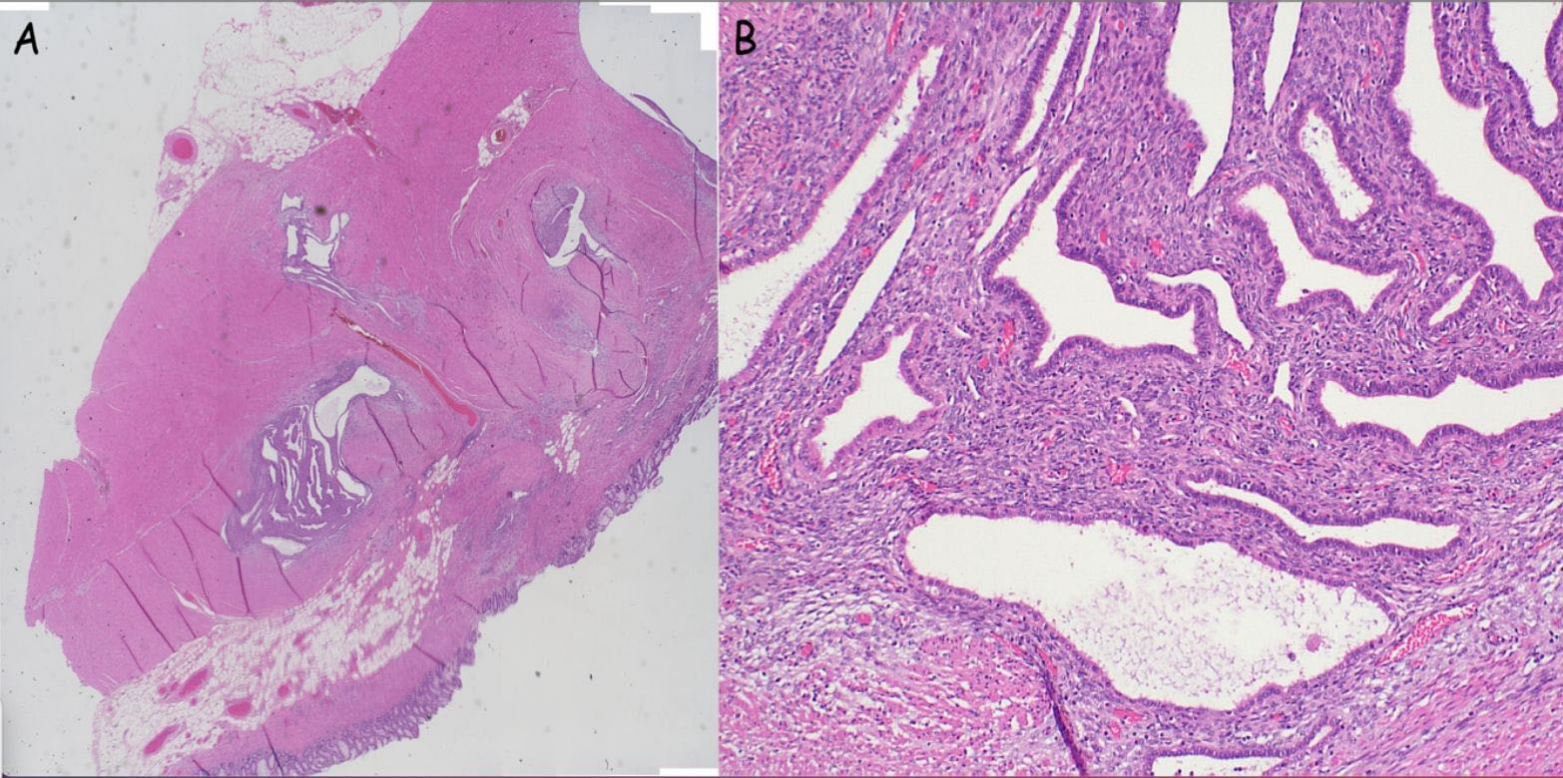
**(A, B)** Bowel wall with evidence of endometriosis (stroma and glands) within the wall (**A**: low power, **B**: High power).

**Figure 3:**
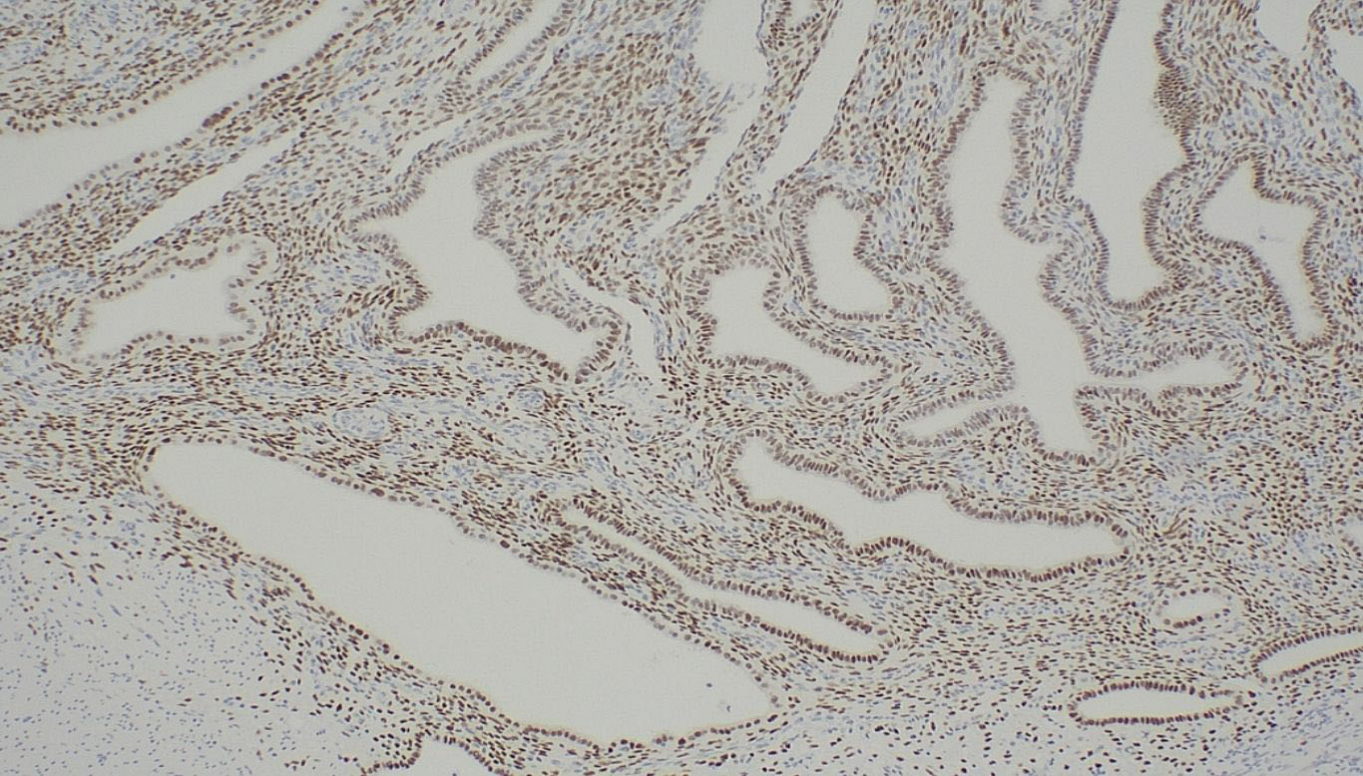
Oestrogen receptor immunohistochemistry; positive staining demonstrated within endometrial stroma and glands.

After a multidisciplinary meeting and discussion with the patient, definitive surgical management was recommended in view of the concerns for underlying malignancy. She proceeded to a laparoscopic total proctocolectomy with end ileostomy in early 2024. Histology demonstrated no active colonic inflammation, however the rectal specimens revealed endometriosis of the submucosa and muscularis propria with muscular hypertrophy corresponding to the stricture (**Figures 2A, B**). Oestrogen receptor immunohistochemistry stained positive in this area, confirming the diagnosis (**Figure 3**).

### 2.4 Outcome and follow-up

Our patient was reviewed in both gastroenterology and colorectal clinics where she is found to be recovering well with resolution of her gastrointestinal and gynaecological symptoms, with specific improvements in her abdominal pain, bloating and dysmenorrhoea.

## 3 Discussion

Endometriosis is a chronic inflammatory condition characterised by ectopic endometrial glands and stroma found outside the uterus, including the pelvis and other extra-pelvic organs (4). It is a common condition with an estimated global prevalence of 10% in reproductive females (5). Ectopic endometrial lesions will either present as either superficial peritoneal, ovarian endometriomas, or deep infiltrating (6). Bowel endometriosis is the most common extra-pelvic site, with an estimated prevalence between 3.8% and 37% of women with endometriosis (7). Of these, 90% involve the recto-sigmoid junction (4). Endometrial glands and stroma are thought to invade the bowel wall from the serosa inward towards the lumen (7, 8). A systematic review of patients undergoing bowel resections for colonic endometriosis demonstrated that of the 1153 patients included, 95% of endometrial lesions invaded the serosa and muscularis propria, 38% percent to the submucosa and 6% to the mucosa (9).

Endometriosis typically presents with symptoms of dysmenorrhoea, dyspareunia and infertility; however bowel endometriosis can present with these along with non-specific gastrointestinal symptoms including abdominal pain, bloating, diarrhoea, constipation and rectal bleeding (4, 7). Rarely, it may cause bowel obstruction (10). Our patient was not known to have endometriosis, however she reported dysmenorrhoea, intermittent menorrhagia, along with occasional abdominal pain and bloating without evidence of diarrhoea, constipation or rectal bleeding. This creates a diagnostic challenge as both conditions have symptoms which overlap. Recent studies have identified a potential association between IBD and endometriosis. A systematic review reported that the prevalence of endometriosis among those with IBD ranging from 2-3%, notably higher than the 1% observed in control groups. Additionally, in a separate case-control study, 71% of IBD patients had a concomitant diagnosis of endometriosis with compatible symptoms (11, 12). This substantial overlap leads to delays or misdiagnosis. Nonetheless, both gastroenterologists and gynaecologists should consider concomitant disease by obtaining thorough histories and screening for crossover symptoms prompting the need for further investigations or specialist referral. To date no treatments are available that can simultaneously address IBD and endometriosis (12).

Differentiating between a stricture caused from CRC and a stricture caused by endometriosis can be done through imaging and histology. The gold standard for diagnosis of endometriosis is through laparoscopic visualisation of endometrial lesions with histopathological confirmation (13). More recently guidelines have changed to establish a diagnosis through symptomology and imaging with transvaginal ultrasound and if required pelvic magnetic resonance imaging (13). Colonic strictures result from an aberrant cycle of active inflammation and healing which leads to scar tissue formation. They are typically classified as either benign or malignant. Benign strictures can be secondary to IBD, diverticulosis, ischaemia or radiation (14). Rarer causes include cytomegalovirus colitis, tuberculosis, amoeba, amyloidosis, and endometriosis (15). With respect to malignant strictures, in a retrospective study looking at IBD patients undergoing surgery for colonic strictures 3.5% were found to have dysplasia or cancer (16). Our patient’s initial endoscopic stricture biopsy did not demonstrate dysplasia or endometriosis. This likely occurred as endometrial lesions rarely extend past the muscularis propria layer and endoscopic biopsies only obtain superficial sampling. An alternative consideration would be the use of transabdominal ultrasound to aid in differentiating between IBD and endometriosis, however this still requires further validation. In a similar case report, a diagnosis of endometriosis was made through ultrasound guided fine needle biopsy in a pre-menopausal woman with sigmoid colon obstruction with a background of IBD who similarly had negative colonic biopsies for endometriosis (17).

Yet, our patient had a further increased malignancy risk due to the presence of two high-risk features including PSC and presence of an undifferentiated colonic stricture (17). Our multidisciplinary team came to a conclusion that the risk of malignancy in a patient with a recurrent strictures who had UC and PSC was too great to avoid colectomy and further investigations may have led to diagnostic delay of malignancy. Endometriosis of the bowel can be managed with hormonal or surgical management, often surgical management is necessary as hormonal therapy does not provide disease eradication (18, 19). Laparoscopic surgery or bowel resection is offered based of symptoms and location of disease (18).

The importance of IBD dysplasia screening cannot be overstated and development of surveillance programs in IBD patients ultimately reduces morbidity and mortality (1). Our patient was a part of an annual screening program due to concomitant PSC in line with the current European Crohn’s Colitis Organisation (ECCO) and American Gastroenterological Association (AGA) guidelines (1, 2). If dysplastic lesions are detected, potential management options include endoscopic or surgical resection and surveillance (1).

This case presents a challenging scenario of managing rectal strictures in IBD patients with high risk factors for malignancy and the importance of recognising alternative causes of bowel strictures. In our case, the rectal stricture was found to be a benign stricture secondary to endometriosis.

## 4 Learning points/take home messages

1. Vigilant dysplasia surveillance for patients with IBD and other high-risk features is recommended.
2. Alternative aetiologies for colonic strictures should always be considered.
3. There is considerable symptoms overlap between bowel endometriosis and IBD, hence thorough gynaecological history should be considered in those with non-specific symptoms.

## Data Availability

The original contributions presented in the study are included in the article/supplementary material. Further inquiries can be directed to the corresponding author.
